# Measuring and Overcoming Limits of the Saffman-Delbrück Model for Soap Film Viscosities

**DOI:** 10.1371/journal.pone.0121981

**Published:** 2015-03-30

**Authors:** Skanda Vivek, Eric R. Weeks

**Affiliations:** Department of Physics, Emory University, Atlanta, Georgia, United States of America; University of Manchester, UNITED KINGDOM

## Abstract

We observe tracer particles diffusing in soap films to measure the two-dimensional (2D) viscous properties of the films. Saffman-Delbrück type models relate the single-particle diffusivity to parameters of the film (such as thickness *h*) for thin films, but the relation breaks down for thicker films. Notably, the diffusivity is faster than expected for thicker films, with the crossover at *h/d* = 5.2 ± 0.9 using the tracer particle diameter *d*. This indicates a crossover from purely 2D diffusion to diffusion that is more three-dimensional. We demonstrate that measuring the correlations of particle pairs as a function of their separation overcomes the limitations of the Saffman-Delbrück model and allows one to measure the viscosity of a soap film for any thickness.

## Introduction

Soap films are thin liquid films, stabilized by two surfactant layers on either side. Soap films have complex hydrodynamics [1] that have been widely investigated as early as the late 19th century for example by Plateau [2] and Gibbs [3]. Previous experiments have demonstrated that thin soap films behave in many respects as two-dimensional (2D) fluids [4–8]. Soap films have applications as a wide range of model systems. For example, soap films share similarities to cell membranes [9]. Soap films were used to study swimming fish and flapping flags in a two dimensional wind [7]. Quickly flowing soap films also served as model systems for 2D turbulence [8, 10, 11], which is relevant in our atmosphere at large scales.

We are interested in understanding soap film hydrodynamics using microrheology, by placing tracer particles and analyzing their diffusive motion [4–6]. Particle motions in a soap film are constrained in the third direction, due to small film thickness. Hence, their diffusive motion is two-dimensional and controlled by an effective 2D viscosity of the soap film, *η*_2*D*_. *η*_2*D*_ is expected to be related to the film thickness and other details of the soap solution using the 1957 Trapeznikov approximation [12]. In 1975 Saffman and Delbrück argued that diffusive motion in a fluid membrane is also influenced by the surrounding three-dimensional (3D) viscous fluids with viscosity *η*_3*D*_ on either side of the membrane [13]. In the case of interest to soap films, the surrounding 3D fluid is air with viscosity *η*_3*D*_ = *η*_*air*_. The Saffman-Delbrück approximation [13, 14] relates the observable single particle diffusivity *D* to *η*_2*D*_, *η*_*air*_, and the particle diameter *d*, allowing one to determine *η*_2*D*_ by observing tracer particle trajectories [4].

The Saffman-Delbrück theory is based on modeling a spherical tracer as a cylinder with diameter *d* and height *h*. This seems plausible for spheres with *d* ≥ *h*, but it is questionable how sensible this is for spheres with *d* < *h*. There has only been minimal experimental exploration of this limit [4]. In prior work, it was demonstrated that the Saffman-Delbrück approximation works well up to *h*/*d* = 4, and is no longer applicable for *h*/*d* ≥ 10 [4]. Unfortunately, these experiments did not have data in the interesting regime 4 ≤ *h*/*d* ≤ 10. For their thin films *h*/*d* < 4, they found that diffusive measurements interpreted with the Saffman-Delbrück approximation led to results in agreement with the prediction of Trapeznikov [12] for *η*_2*D*_. Due to the lack of data in the crossover region, it was unclear if the crossover from 2D to 3D behavior was a smooth function of *h*/*d* or some other parameter.

In this work, we present new experimental data of the diffusivity of particles in soap films, to examine more closely the breakdown of the cylindrical assumption of the Saffman-Delbrück approximation. In this current work we use a more reliable means of measuring film thickness *h* and due to experimental improvements are able to get data from a wider range of film thicknesses and particle sizes. While the previous work probed a single *d* at the crossover, in this work we use particles of two different *d* and verify that *h*/*d* is the correct parameter indicating the crossover. We find that the Saffman-Delbrück model works well up to *h*/*d* > 5.2±0.9, and that there is a smooth crossover from thin films to thick films as a function of *h*/*d*. We additionally examine the correlated motion of pairs of particles as a function of their separation to independently infer *η*_2*D*_, and in doing so we demonstrate that the Trapeznikov prediction is valid for all soap films, independent of *h*/*d*. Our results clarify that this correlated particle motion is a more effective way to measure *η*_2*D*_ from observing diffusive motion of tracers in a soap film. While this had been suggested by prior work [4], our results provide a wider range of data in support of the interpretation put forth earlier.

Further background discussion of the Saffman-Delbrück and Trapeznikov approximations is presented in the following section.

## Hydrodynamic theory

### Single particle diffusion in thin films

Our starting point for diffusion is to measure the mean square displacement of tracer particles, which is related to the diffusion constant as

$$\begin{array}{c} \frac{\left\langle \Delta r^{2}\right\rangle }{4\tau }=D_{1p} \end{array}$$ (1)

Here *τ* is the lag time for the displacement, $\Delta r=|\vec{r}\left(t+\tau \right)-\vec{r}\left(t\right)|$, and the subscript 1*p* indicates that this diffusion constant *D*_1*p*_ is based on averages over single particle motion.

In 3D, the single particle diffusion constant relates to the 3D viscosity *η*_3*D*_ by the Stokes-Einstein-Sutherland equation [15, 16]

$$\begin{array}{c} D_{B}=\frac{k_{B}T}{3\pi \eta_{3D}d}, \end{array}$$ (2)

with Boltzmann constant *k*_*B*_, absolute temperature *T*, and particle diameter *d* the subscript *B* indicates this is a bulk diffusivity. However, this equation does not apply to soap films, for several reasons. First, as noted previously, 2D fluid flows are fundamentally different than 3D flows, leading to more complex relationships between *D*_1*p*_ and *d*. Second, diffusion and flow in a soap film is influenced by the viscosity of the surrounding air. Third, the viscosity in 2D has different units: Pa⋅s⋅m as compared to Pa⋅s in 3D. In 1975 Saffman and Delbrück treated this case, deriving an approximation for *D*_1*p*_ for the situation of a 2D membrane with interfacial viscosity *η*_2*D*_ with fluid of 3D viscosity *η*_3*D*_ on both sides of the membrane [13]. Using the small parameter *ε* = *dη*_3*D*_/*η*_2*D*_, Saffman and Delbruck found [13, 17]

$$\begin{array}{c} D_{1p}=\frac{k_{B}T}{4\pi \eta_{2D}}\left[\ln\left(\frac{2}{\epsilon }\right)-\gamma_{E}\right], \end{array}$$ (3)

using Euler’s constant *γ*_*E*_ = 0.577. In particular, this derivation treated the 2D membrane as a thin 3D layer of fluid with 3D (“bulk”) viscosity *η*_*B*_, thickness *h*, and therefore a 2D viscosity *η*_2*D*_ = *hη*_*B*_. They considered the diffusion of disks of diameter *d* and height *h* which spanned the membrane thickness, and which only move horizontally (within the membrane). Eq 3 works well for small *ε* (large *η*_2*D*_). *ε* ^−1^ is often termed the Boussinesq number Bo, so equivalently Eq 3 works well for large Bo. [18, 19]. Hughes *et al*. [20] extended Eq 3 to work for all *ε*, with a useful approximation to their result added later by others [21, 22]. While the large *ε* limit is of less interest to small particles diffusing in soap films, we note that the large *ε* behavior has been experimentally verified in lipid membranes [22] and liquid crystal films [23]. Note that Eq 3 assumes that the interface horizontal extent *R* is sufficiently large (*d*/*R* < *ε*), which is the case for us (*R* ≈ 1 cm) [13].

Soap films are made from a regular fluid with added surfactant molecules, and it is straightforward that the effective viscosity *η*_2*D*_ for a soap film should depend on its constituents. This was first described in 1957 by Trapeznikov [12]. He noted that there should be a contribution *hη*_*B*_ from the bulk fluid used to make the soap film. Dimensionally, this makes sense, and it is also physically reasonable that *η*_2*D*_ should increase for larger *h* or *η*_*B*_. Trapeznikov also noted that the surfactants at the fluid-air interface should themselves act like a 2D fluid and contribute their own 2D viscosity *η*_*int*_, so therefore the effective 2D viscosity of the entire soap film would be given by

$$\begin{array}{c} \eta_{2D,T}=\eta_{B}h+2\eta_{int}. \end{array}$$ (4)

This then is a prediction that *η*_2*D*_ measured using Eqs 8 and 3 is equal to *η*_2*D*,*T*_. This prediction was confirmed in prior experiments by Prasad and Weeks for thin soap films with *h*/*d* < 7±3 [4, 5], but for thicker films diffusion seemed to sense the 3D nature of the film and follow more closely Eq 2 [5].

### Two particle correlated motion in thin films

Two-particle microrheology is an alternative analysis technique that complements measuring single-particle diffusion via Eq 8 [24, 25]. Conceptually, this examines correlations between the motion of each pair of particles. If our soap films obey 2D hydrodynamics, two-particle correlations should obey 2D hydrodynamic theory [26] in which the correlations decay as ln(*R*), where *R* is the separation between two particles. This is in contrast to the situation in 3D, in which correlations decay as 1/*R* [24].

Specifically, there are four eigenmodes corresponding to pairwise motion in 2D. Two of these modes are parallel motions (+) in the longitudinal (*x*) direction (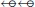) and transverse (*y*) direction (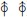). The other two are anti parallel motions (-) along *x* (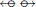) and *y* (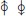). These four correlation functions are calculated by:

$$\begin{array}{ccc} D_{x\pm }\left(R,\tau \right) & = & {\left\langle \frac{1}{2}{\left[x_{i}\left(\tau \right)\pm x_{j}\left(\tau \right)\right]}^{2}\delta \left(R-R_{ij}\right)\right\rangle }_{i\neq j}\\ D_{y\pm }\left(R,\tau \right) & = & {\left\langle \frac{1}{2}{\left[y_{i}\left(\tau \right)\pm y_{j}\left(\tau \right)\right]}^{2}\delta \left(R-R_{ij}\right)\right\rangle }_{i\neq j} \end{array}$$ (5)

Where *i* and *j* denote different particles and *R*_*ij*_ denotes particle separation. For a purely viscous system, much as ⟨Δ*r* ^2^⟩ ∼ *τ* (e.g., Eq 1), these correlation functions also will be proportional to the lag time *τ*.

Di Leonardo *et al*. proposed a theory [26] based on the two-dimensional Stokes equation. The theory makes several approximations: neglecting stresses from the bounding fluid (air), neglecting the finite film size (in the lateral dimension), and neglecting inertia. The Oseen tensor is obtained from the two-dimensional Stokes equation from which the four eigenvalues corresponding to the eigenmodes given above can be solved [26]. The solutions find correlations depending on *R* as:

$$\begin{array}{ccc} D_{x\pm }/\tau & = & A\pm B\ln\frac{L}{R}\\ D_{y\pm }/\tau & = & A\pm B\left(\ln\frac{L}{R}-1\right) \end{array}$$ (6)

with

$$\begin{array}{ccc} A & = & 2D_{1p}\\ B & = & \frac{k_{B}T}{2\pi \eta_{2D}}. \end{array}$$ (7)

*L* is a length scale beyond which the approximation fails, although it can fail for different reasons in different situations. For example, similar to the Saffman-Delbrück approximation, *L* could be related to the smaller of the system size *R* and the Saffman length *l*_*S*_ = *η*_2*D*_/2*η*_3*D*_ [26]. To be clear, the results of Eq 6 are based on a purely 2D model of hydrodynamic correlations, without any 3D fluid present.

Note that in Ref. [26], they assumed *η*_2*D*_ = *hη*_*B*_ for a soap film [26], but it has been demonstrated that *η*_2*D*_ = *η*_2*D*,*T*_ is more appropriate for soap films [4]. As can be seen in Eq 4, *η*_2*D*,*T*_ ≈ *hη*_*B*_ for thick films where *h* is large, so the distinction only matters for thin films.

In summary, measuring the correlations described in Eq 5, fitting to Eq 6, and interpreting the fit parameters with Eq 7 is another route to measuring *η*_2*D*_. One advantage of this method is that it should be less sensitive to the exact position of small tracer particles within the film: partially protruding into the air, or fully immersed in the film. Protrusion of a tracer particle into the air certainly affects its single-particle mobility [27], and so using single-particle analysis methods may result in errors in determining *η*_2*D*_. In contrast, the two-particle correlations are measuring long-range hydrodynamic correlations which are insensitive to the local details [24]. Even if one examines correlations between one particle protruding through the soap film surface and a second particle fully immersed in the film, the particular motions due to the local environment of each particle will be uncorrelated, and the long-range correlations should feel only the hydrodynamic effects of the soap film itself (perhaps as modified due to coupling with the air). Also, the two-particle correlation predictions (Eq 6) do not make any assumptions about the nature of the tracer particles, but focus only on hydrodynamics. Historically, this insensitivity to the tracer details was a key strength and motivation for two-particle correlation techniques in soft matter [24]. In other words, these predictions do not assume that the tracers are embedded cylinders, unlike the Saffman-Delbrück approach. So, these predictions should hold even in the limit of small tracer particles with diameters smaller than the film thickness, *d* ≪ *h*. Another way to see the advantage of the method is that the correlations between two particles separated by a distance *R* is probing the same hydrodynamics as a particle of size *R* would see, and this overcomes the concerns of small particle size [24].

## Materials and methods

### Samples and data acquisition

**Fig 1:**
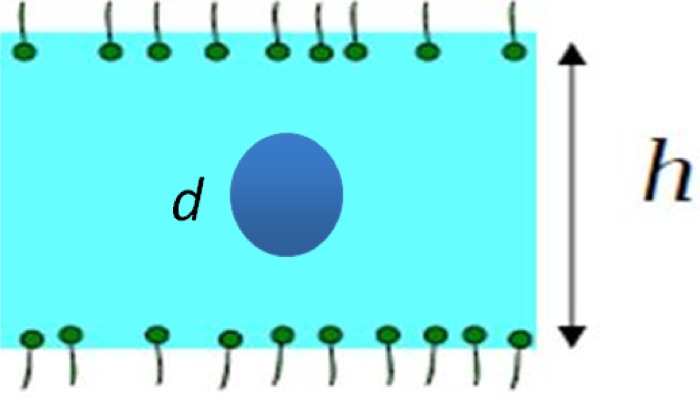
Cartoon depicting soap film of thickness *h*, with a single representative particle of diameter *d* < *h*. On both air-film interfaces, representative soap molecules are shown. As discussed in the text, we believe it is likely that particles with *d* < *h* sit in the interior of the film, but we cannot rule out the possibility that some particles are trapped at the air-film interface.

We make our soap films from bulk solutions of water, glycerol, and surfactant. We use the dishwashing detergent Dawn as our surfactant to stabilize the interfaces of the soap film. Once the bulk solution is prepared, we add fluorescent polystyrene particles of known diameter (we use *d* = 0.1, 0.18, and 0.5 *μ*m). We then draw a soap film from the bulk solution using a rectangular metal wire frame with dimensions ≈ 1.5 cm×2.0 cm, and thickness *h*. A cartoon depicting the film and representative particle is shown in Fig 1.

We have a microscope chamber made with a water filled sponge lining to increase humidity and reduce evaporation of water from the soap film. This chamber is mounted on our upright microscope, and the freshly drawn soap film is placed inside the chamber. We seal the chamber as far as possible from outside air, to reduce convection at the soap-film interface.

We use fluorescence microscopy to record a 33 s movie of particles diffusing in the soap film at a frame rate of 30 images/s. The film is illuminated using an arc lamp, and a movie of particles diffusing is taken using a CCD camera. Microscope objectives 20×, 40×, and 63× are used for particles of diameter *d* = 0.5, 0.18, and 0.1 *μ*m respectively. We post-process the movies using particle tracking algorithms [28] to extract particle positions from individual frames. We note that there is position uncertainty from particle localization accuracy and camera motion blur due to the finite shutter speed. There are multiple ways of measuring this uncertainty [29–31]. We use the CVE method [31] to estimate this uncertainty. The 3 different particle sizes of diameters *d* = 0.1, 0.18, and 0.5 *μ*m have uncertainties of 0.10, 0.065, and 0.080 *μ*m respectively.

Some macroscopic flow of the soap film in its frame is unavoidable, resulting in coherent flow of the tracers in our movies. Between each video frame we compute the center of mass motion by finding the average displacement of every particle. The uniform flow is then subtracted from the particle positions to provide their relative locations in the frame of reference co-moving with the flow. This lets us then study the diffusive motion of the individual particles. However, drift removal may artificially reduce true long-range hydrodynamic correlations. We have checked that our algorithm does not unduly affect the correlations; details of this are given in the S1 Appendix. The raw movies are available as supplemental online material S1 Movie, S2 Movie, and S3 Movie.

We would like to know where our particles are within the soap film, but this is difficult to determine directly given that the depth of focus of our microscope is comparable to the soap film thickness. For particles in films thinner than the particle diameter, it is highly likely that capillary forces ensure that the particle lies symmetrically within the film [32]. One experiment demonstrated that pinning of the contact line at a rough particle surface can sometimes delay reaching the equilibrium position for time scales longer than our experiments [33], and we cannot rule out that our particle positions may not be equilibrated. For particles in films thicker than their diameter, particles might sit at the air-water interface to reduce the air-water surface energy. However, as mentioned in a prior study of soap films [5] and as we observe, small particles in very thick films diffuse as if they are in a bulk solution of the soap film liquid. This would not happen for particles trapped at an interface [27]. The two-particle correlation functions should be less sensitive to the exact positions of the tracer particles. Note that a recent paper showed comparisons between microrheology and macrorheology results, and pointed out that measurements of particles not in the interface are misleading [19]. They were studying cases where the interface was with a bulk liquid and air, quite different from our finite-thickness films. Our particles should still be reporting film properties no matter their location. Our results for the two-particle correlations support this, as will be described in our Results and Discussion section.

### Measuring soap film thickness

After taking the movie, we take the film out of the microscope humidity chamber and measure the film’s thickness using the infrared (IR) absorption of the water based soap films at wavelength *λ* = 3.0 *μ*m. This is based on a previously established technique [34] which we briefly summarize here. Light is incident on the soap film from an incandescent lamp. The light passes through an optical chopper, to chop the light at a particular frequency. This light is then focused on the soap film by an IR lens to a spot size of ∼ 2.5 mm. An IR filter (3.00±0.01 *μ*m JML Optical Industries, LLC) in the beam path allows only wavelength of 3.0 *μ*m to pass through. Finally, the light is refocused on an InAs photodiode detector (Teledyne Judson, model J12TE2-66D-R01M) by a second IR lens. The signal from the photodetector is obtained from a lock-in amplifier (Signal recovery, model 7265) locking with the external reference frequency of the chopper, which reduces noise. We separately measure the refractive index and absorption coefficient of each bulk solution at the same wavelength. From measured transmittance through the film and these details of the bulk solution, we calculate the film thickness using a modified Beer-Lambert law that takes into account the multiple reflections in the soap film. This method is slightly different from prior work [4], and is a notable improvement in that the thickness measurement is done physically adjacent to the microscope and thus is done within 30 s of taking the microscopy data, allowing for higher accuracy. In the prior work of our group, the films often popped before their thickness could be measured. Additionally, there was a lag of several minutes between the microscopy movies and the thickness measurement, which potentially allowed for the film to drain or evaporate and thus increased the uncertainty of those film measurements [4].

**Fig 2:**
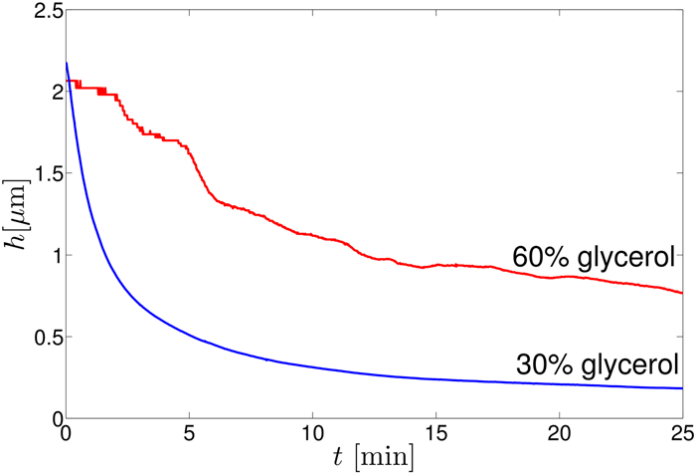
Thickness as a function of time, which decreases due to soap film drainage, for films with weight percent glycerol as indicated.

Soap films thin over time at the center due to drainage of liquid towards the sides arising from capillary forces. Fig 2 shows soap film drainage for 30 percent and 60 percent glycerol weight content soap films. In general, we observe that films made of bulk solutions with lower glycerol concentration drain faster than those made of higher glycerol concentration.

The timing of our experiment matters. When the soap film is initially drawn on to our frame, in addition to the drainage, there are also transient flows primarily due to air currents. Accordingly, after placing the film into the microscope chamber and sealing the chamber, we wait for 10 to 20 minutes before taking the movie. This allows the initial rapid drainage to slow, and also the transient flows. The duration of the movie (33 s) and delay before measuring the thickness (30 s) are short on the time scale of the drainage, as is apparent from Fig 2.

In our soap film thickness measurement, there are three main sources of error. The first source of error is due to soap film drainage in the time between our microscopy and soap film thickness measurements. This error is higher in low viscosity soap films due to their faster drainage as mentioned above. We quantify this error by measuring the maximum change in thickness during 30 s from the curves shown in Fig 2, which is a maximum of 0.03 *μ*m. The second source of error comes from the precision of the lock-in amplifier and noise present in the measurement, including possible variations in illumination. This is worse for thicker films (which have less transmitted light) and is at most 0.02 *μ*m. The third source of error is from residual soap film flows and the fact that soap films are not uniformly thick. This error is quantified by the fluctuations in the soap film thickness after long times, when film thickness reaches equilibrium, i.e., when thickness is almost constant. This error is negligible for low viscosity films, but plays an effect in films of higher viscosity, the maximum error being 0.02 *μ*m. We assume these errors are additive, so for thick films, our thickness measurement uncertainty is ±0.05 *μ*m.

## Results and Discussion

### Single particle diffusion

In the prior soap film studies by our group [4, 5], the single particle diffusion constant was measured through the mean square displacement of tracer particles from Eq 1—that is, plotting ⟨Δ*r* ^2^⟩ as a function of *τ* and fitting a line to find *D*_1*p*_. A recent paper by Vestergaard *et al*. shows that this is not the best method [31]. In the current study, we use their covariance based estimator (CVE) method. This yields the optimal *D*_1*p*_ from

$$\begin{array}{c} \frac{\left\langle \Delta r_{n}^{2}\right\rangle }{4\Delta t}+\frac{\left\langle \Delta x_{n}\Delta x_{n+1}\right\rangle }{2\Delta t}+\frac{\left\langle \Delta y_{n}\Delta y_{n+1}\right\rangle }{2\Delta t}=D_{1p} \end{array}$$ (8)

Here Δ*r*_*n*_ denotes displacement between frames and *n* denotes frame number. *x* and *y* denote the two dimensions, and Δ*t* is the frame rate of recording, 33 ms. The averages are taken over all video frames *n* and all particles. This method has the advantage of removing fictitious correlations between subsequent frames which can be due to video artifacts or other subtle experimental errors [31].

**Fig 3:**
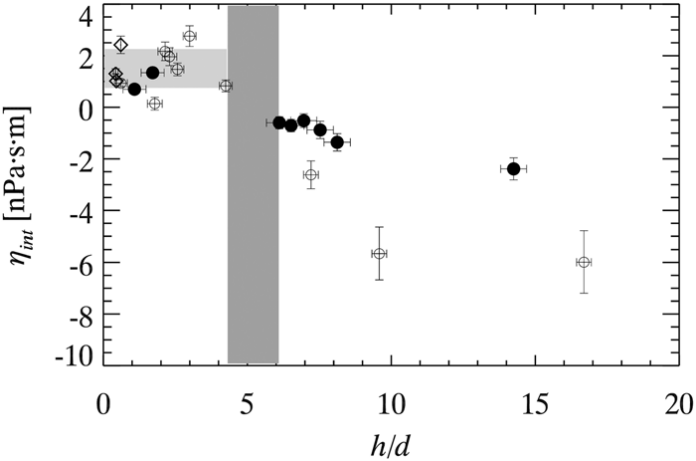
Plot of interfacial viscosity from single particle diffusion measurements as a function of *h/d*. Filled circles denote particles of diameter 0.1 *μ*m, open circles denote particles of diameter 0.18 *μ*m and diamonds denote particles of diameter 0.5 *μ*m. The horizontal light shaded region represents *η*_*int*_ = 1.42±0.74 nPa⋅s⋅m based on the mean and standard deviation of the data for *h*/*d* < 5. The vertical dark shaded region represents the crossover from physical behavior at small *h*/*d* to unphysical behavior at *h*/*d* > 5.2±0.9. The horizontal error bars are due to uncertainties of *h*, and vertical error bars are due to uncertainties of *h* and *η*_2*D*_ (see Eq 4).

In a bulk (3D) liquid, diffusivity *D*_*B*_ is a constant given by Eq 2 [15, 16]. However, in soap films we expect diffusivity *D*_1*p*_ to follow Eq 3 using *η*_2*D*_ equal to the Trapeznikov viscosity, *η*_2*D*,*T*_ given by Eq 4. Reversing this logic, we compute *η*_2*D*_ from measured single particle diffusivity *D*_1*p*_ using Eq 3, the known viscosity of air (*η*_*air*_ = 0.017 mPa⋅s), and the known tracer diameter *d*. We then obtain *η*_*int*_ from this measured *η*_2*D*_ via Eq 4. This interfacial viscosity should be independent of film thickness as it is a property solely of the soap-air interface, and the soap concentration is kept constant throughout our experiments. This conjectured independence is a test of the approximations, and accordingly in Fig 3 we show *η*_*int*_ as a function of *h/d*. Each data point corresponds to a particular soap film. For *h*/*d* < 5, *η*_*int*_ is positive and roughly constant; in this region, the approximations of the Saffman-Delbrück model work well. Taking the mean value of the data for *h*/*d* < 5 gives us *η*_*int*_ = 1.42±0.74 n⋅Pa⋅s⋅m. This agrees with a previously published value of 0.97±0.55 n⋅Pa⋅s⋅m for soap films made with the same surfactant [5]. While we do not have a direct method to measure viscosity of the soap-air interface, the rough agreement of the measurements for *h*/*d* < 5 seen in Fig 3 demonstrate that single-particle diffusivity is one method to measure *η*_*int*_ for a soap film, as has been argued previously [5].

Fig 3 also shows that for larger *h*/*d*, *η*_*int*_ is unphysically negative and also quite variable. The crossover occurs at *h*/*d* = 5.2±0.9. This value is obtained from the gap in our data in the crossover region, i.e. 5.2 denotes the center of the gap with a width of 0.9 on either side. Our current observation is an improvement over prior experiments which had a larger gap and identified the crossover as *h*/*d* = 7±3 [4]. Furthermore, the reasonable agreement for this crossover location between the different particle sizes (different symbols in Fig 3) is good evidence that the crossover is indeed a function of *h*/*d*. As noted in the Hydrodynamic theory section, a breakdown for large *h*/*d* is expected. The Saffman-Delbrück approximation treats the tracer as a cylinder with height equal to the film thickness, which is dubious for *h*/*d* > 1. Given that, it is remarkable that this approximation holds up to *h*/*d* = 5.2±0.9. Another way to state that is, given our experimental uncertainty, we see no deviations from 2D behavior up to *h*/*d* = 4.3.

For larger *h*/*d*, the interfacial viscosities in Fig 3 are unphysically negative, showing that particles are diffusing faster than expected—that is, faster than one expects, if *η*_2*D*_ were equal to *η*_2*D*,*T*_. We also observe that particles of smaller diameter are less negative in Fig 3. This is because for large *h*/*d*, particles diffuse more like in bulk [35], i.e., measured diffusion *D*_1*p*_ ≈ *D*_*B*_, as in Eq 2. Equating this *D*_1*p*_ to the Saffman-Delbrück equation Eq 3 and approximating the ln term as a constant, we get for large *h*/*d* that the measured *η*_2*D*_ ∼ *η*_*B*_ *d*. Using Eq 4 to extract *η*_*int*_ from these apparent *η*_2*D*_ values, we see that the particular negative values for *η*_*int*_ will be smaller in magnitude for smaller *d*, and that the specific values will also depend on *η*_*B*_ (which differs from film to film in our experiments). The differing *d* and *η*_*B*_ give rise to the scatter of the data seen in Fig 3 for *h*/*d* > 5.2. Despite the scatter, it is apparent that there is a fairly smooth crossover from the regime where the Saffman-Delbrück approximation works to where it fails.

### Two particle correlated motion

**Fig 4:**
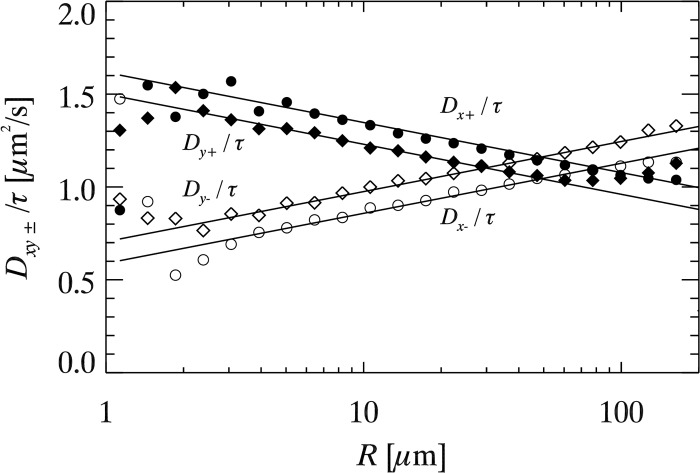
Two particle correlations in a single soap film measurement as a function of particle separation *R*. Particles of diameter *d* = 0.18 *μm* were used, and soap film thickness was *h* = 0.46±0.04 *μm*. The solid lines are fits from Eq 6 with *A* = 1.09 *μ*m^2^/*s*, *B* = 0.12 *μ*m^2^/*s* and *L* = 81 *μ*m. The data are computed from all particle pairs and averaging over a wide range of lag times *τ*.

The two-particle measurements should not suffer from the difficulties the one-particle measurements have, as the two-particle correlations reveal the long-range hydrodynamic correlations of a soap film rather than the diffusive properties of a single particle. As described by Eq 5, we compute the two-particle correlation functions for a particular experiment and plot them in Fig 4 averaging over a wide range of lag times *τ*. The data behave as expected. For example, for nearby particles (small *R*), particles move in similar directions and the parallel correlations are large (*D*_*x*+_ and *D*_*y*+_, indicated by the solid symbols). The anti parallel motions are smaller for small *R* (*D*_*x*−_ and *D*_*y*−_, indicated by the open symbols). All of the correlation functions vary logarithmically with *R*, and Eq 6 fit the data well as seen by the lines. These lines have three fitting parameters, *A*, *B*, and *L*, which have a simple graphical interpretation. *A* denotes the point of intersection of positive and negative correlations on the vertical axis, *B* is the slope of the lines, and *L* is the point of intersection of *D*_*x*+_ and *D*_*x*−_ on the *R*-axis.

**Fig 5:**
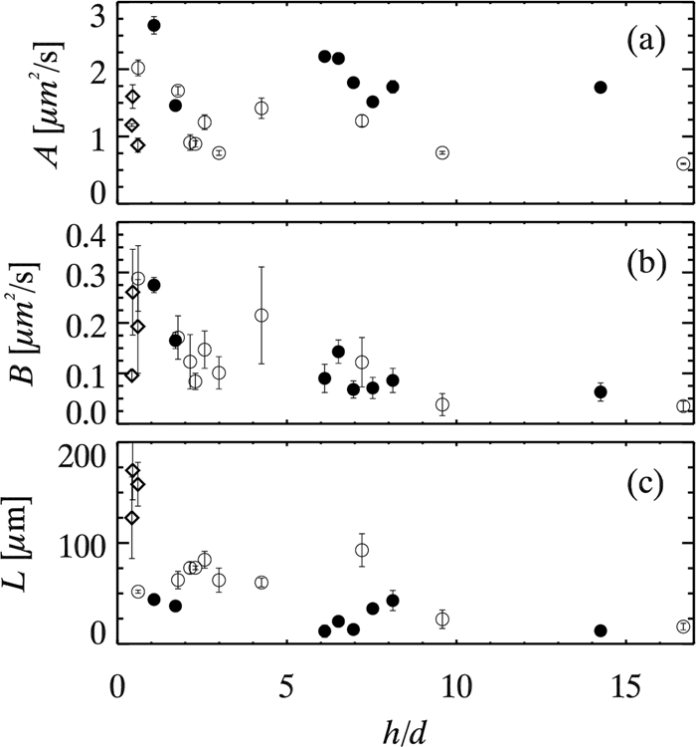
Fit parameters for all experiments as a function of *h*/*d*. Symbols denote particle diameters as in Fig 3. See Eqs 6 and 7 for the meaning of the fit parameters. The vertical error bars are from the standard deviations of each fit parameter calculated for the different *τ*’s.

**Fig 6:**
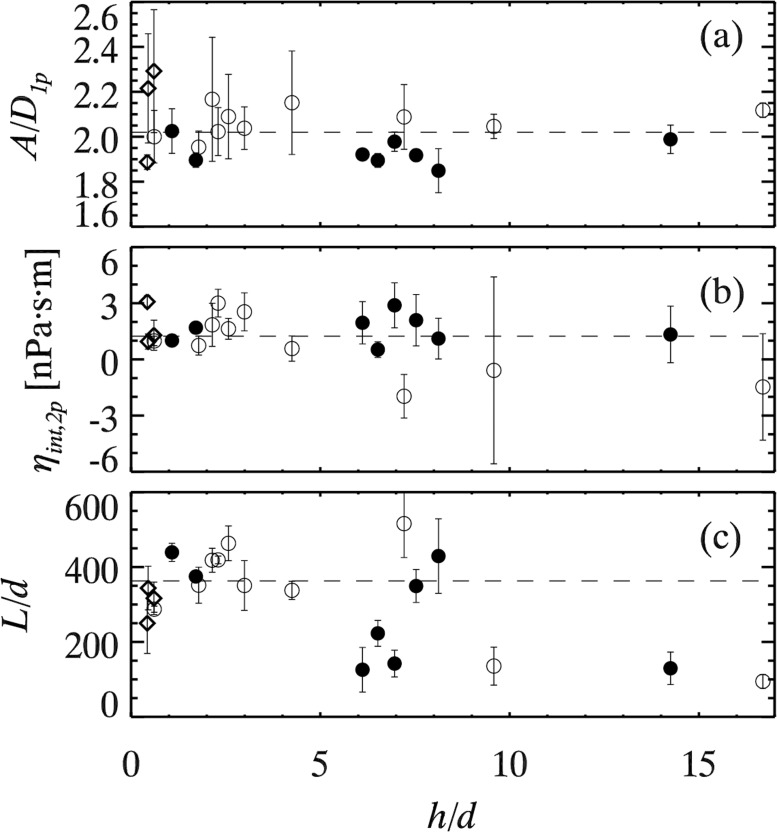
Fit parameters for all experiments as a function of *h*/*d*. Symbols denote particle diameters as in Fig 3. (a) *A*/2*D*_1*p*_ is roughly constant, with mean value 2.02±0.12 over all data as indicated by the dashed line. (b) $\frac{1}{2}[\frac{k_{B}T}{2\pi B}-\eta_{B}h]$, which should theoretically be *η*_*int*_. The dashed line shows the mean value *η*_*int*,2*p*_ = 1.20±1.30 nPa⋅s⋅m. (c) *L*/*d*, where the dashed line represents the mean value *L*/*d* = 360±60 for the data with *h*/*d* < 5.2.

For each experimental movie, we compute Eq 5 as a function of *τ*, and fit data for each *τ* to determine coefficients *A*(*τ*),*B*(*τ*), and *L*(*τ*). These have a slight *τ* dependence, but minimal enough that we simply average over several exponentially-spaced values of *τ*; see S1 Appendix for further discussion. We refer to the *τ*-averaged values as *A*, *B*, and *L* for the remainder of this paper. We plot these fit parameters in Fig 5 as a function of *h*/*d*. The effects of collective drift subtraction on our results are discussed in detail in the S1 Appendix. The uncertainties are reflected by the error bars shown in Fig 5. The data are scattered, which is to be expected as the parameters depend on far more than *h*/*d*. The different data correspond to a variety of bulk viscosities and particle sizes. Accordingly, we rescale each of these to make sense of their behavior, and show the rescaled results in Fig 6. We now discuss these rescaled results.

From Eq 7, we expect *A* = 2*D*_1*p*_ where *D*_1*p*_ is the single particle diffusivity of that measurement. Fig 6(a) shows *A*/*D*_1*p*_ is constant with value 2.02±0.12, in great agreement with the theoretical prediction. Previous work by Prasad and Weeks found a value of 1.9±0.1, a moderately concerning discrepancy from the theoretical prediction. The improvement in our current results is due to the CVE method we now use to measure *D*_1*p*_ [31]. If we instead use the prior method of fitting a line to ⟨Δ*r* ^2^⟩ we find the erroneous result *A*/*D*_1*p*_ = 1.83±0.09, in agreement with the earlier concerning result.

Fig 6(b) shows $\frac{1}{2}[\frac{k_{B}T}{2\pi B}-\eta_{B}h]$. This quantity should be the interfacial viscosity *η*_*int*_ as seen by rearranging Eq 7. Hence, this is a method to find the interfacial viscosity from two particle correlations. Averaging all the data in Fig 6(b), we find *η*_*int*,2*p*_ = 1.20±1.30 nPa⋅s⋅m. This is consistent with our single particle measurement (*η*_*int*,1*p*_ = 1.42±0.74 nPa⋅s⋅m). While the two-particle measurement has a larger uncertainty, we believe the two-particle value to be more reliable as it uses data from all soap films, both thick and thin. Moreover, this two-particle measurement is robust to concerns about the particle position within the film, as previously discussed. Our value of *A* should depend on the tracer details as it should be tied to *D*_1*p*_ through Eq 7, but *B* and thus *η*_*int*_ should be measuring true properties of the soap film.

Interestingly, rescaling the third fit parameter *L* by the particle diameter *d* plausibly collapses the data, especially for small *h*/*d*, as shown in Fig 6(c). For thin films (*h*/*d* < 5), *L*/*d* = 360±60, indicated by the dashed line. For thicker films *L*/*d* shows scatter and for the most part is smaller than the thin film value.

Di Leonardo *et al*. [26] discuss the possible origins of the length scale *L*. In a purely theoretical infinite-extent planar 2D fluid, there is no cutoff length scale *L*, and correlations die out at infinity. In reality, the finite system size provides one potential cutoff length scale, which was the case in their work with small films. Our film boundary is at least 500 *μ*m away from our field of view, so this seems unlikely to explain our values of *L* of the order of 10-200 *μ*m. Particle motion relative to the film can lead to another length scale [14, 18, 26], but our particles are passive tracers (in contrast to Ref. [26] for example, which used laser tweezers to move particles). Another possibility is stresses from the surrounding air [14, 18, 26], which cannot be neglected at distances larger than the Saffman length *l*_*s*_. In our system, *l*_*s*_ = *η*_*B*_ *h*/2*η*_*air*_, which varies from 10−1000*μ*m. However, our observed *L* does not have such a wide range. Furthermore, our thicker films generally have higher *η*_*B*_ than thinner films, and hence larger *l*_*s*_, yet have smaller values of *L*. None of these length scales seem to match our observed *L*, and these possibilities do not explain our observed dependence of *L* on particle size *d*. Another possible candidate is the capillary interactions between particles. Previous studies found that capillary interactions between particles in a freely suspended liquid film can cause particle-particle interactions even at distances greater than 100*μ*m [32, 36, 37], and these interactions should scale with *d*. These forces would depend on if particles penetrate zero, one, or two of the film-air interfaces, but capillary forces should not otherwise depend on the film thickness, so the variability seen in Figs 5(c) and 6(c) as *h*/*d* changes seems to contradict this. Additionally, as explained in the Materials and methods section, in thick films we think our particles are likely to be in the film interior—not penetrating either film-air interface—and thus not experiencing capillary forces. One final possibility is that the theory [26] takes only two particles into account. We typically observe *O*(50) particles in a field of view, and perhaps many body effects are present in our data. These might affect *L* by screening particle-particle correlations. These effects however are more complicated to model, and determining a length scale due to many-body effects is not possible. Moreover, our data are from a variety of concentrations all in the fairly dilute limit, and concentration variations seem not to explain the behavior of *L*. For details, S1 Dataset contains a table with all our data including concentrations and fit parameters.

## Conclusions

We have used two different methods for measuring the effective two-dimensional viscosity *η*_2*D*_ of a soap film. The 1957 paper by Trapeznikov [12] put forth Eq 4 conjecturing that this viscosity is related to the soap film thickness, the viscosity of the fluid used to form the film, and a contribution from the surfactant layers bounding the film; in other words, *η*_2*D*_ = *η*_2*D*,*T*_. As we have used the same surfactant concentration for all of our soap films, the validation of our methods for measuring *η*_2*D*_ is the consistency between different measurements of *η*_*int*_, the contribution to *η*_2*D*_ from the surfactant layers. Fig 3 shows that for single-particle measurements, we can get plausible values of *η*_*int*_ for thin films only. Fig 6(b) shows that using two-particle correlations, we get moderately consistent values of *η*_*int*_ from all of our measurements. The scatter of the data in both of these figures shows that neither of these methods are fool-proof, and best results are obtained by averaging over many films. On the other hand, given the variability of our tracer particle size (a factor of 5), bulk viscosity of the soap film solutions (a factor of 4), and film thicknesses *h* (a factor of 30), the agreement of the *η*_*int*_ data is encouraging. Our measurement of *η*_*int*_ = 1.20±1.30 nPa⋅s⋅m based on the two-particle correlations is the value we have the most confidence in, as it uses data from every experiment we have done and is least dependent on the details of the tracer particles.

For larger soap films, the one-particle data of Fig 3 show unphysically negative *η*_*int*_ values, whereas for the two-particle results the data are generally physically plausible [Fig 6(b)]. The one-particle data are due to the breakdown of the assumptions behind the Saffman-Delbrück model, which models the tracers as cylinders which span the soap film thickness. Given this, it is pleasantly surprising that the Saffman-Delbrück approach works for films up to four times thicker than the spherical particle diameter. The two-particle method, in contrast, does not depend on the details of the tracers as sensitively, but rather on the long-range hydrodynamic properties of the soap film mas a two-dimensional fluid. That these hydrodynamic properties indeed behave in a two-dimensional manner is demonstrated in Fig 4 where the two-dimensional theory fits through the data.

These methods for measuring *η*_2*D*_ and *η*_*int*_ should be useful for measuring the shear viscosities of other surfactants. Our confirmation that the flow fields are two-dimensional in character on length scales of 5−100 *μ*m are a useful complement to prior macroscopic experiments that treated soap films as two-dimensional fluids [7, 8, 10, 11]. In summary, the diffusive motion of particles appears quasi-two-dimensional for thin films but not for thick films, whereas the long-range flow fields appear quasi-two-dimensional for both thick and thin films.

## Supporting Information

S1 Appendix Effect of drift subtraction on two particle correlation results. (PDF)

S1 Dataset All Soap film data. (XLS)

S1 Movie Movie of particles of diameter 0.1 *μm* diffusing in a soap film. (AVI)

S2 Movie Movie of particles of diameter 0.18 *μm* diffusing in a soap film. (AVI)

S3 Movie Movie of particles of diameter 0.5 *μm* diffusing in a soap film. (AVI)
