# Supplementary material for: Measuring and Overcoming Limits of the Saffman-Delbrück Model for Soap Film Viscosities
Source: PLoS One. 2015 Mar 30;10(3):e0121981. doi: 10.1371/journal.pone.0121981 (PMC4379181; doi:10.1371/journal.pone.0121981)
Supplement: S1 Appendix — (PDF) [file pone.0121981.s001.pdf]

Effect of drift subtraction on two particle correlation results

Some macroscopic flow of the soap film within the soap film holder is inevitable. This can be quantified by observing that the particles have a slow net drift. That is, $\langle\Delta\vec{r}\rangle = \vec{v}(t)\tau$ with a slowly varying velocity $\vec{v}$, for small lag times τ, and where the angle brackets indicate an average over the displacements $\Delta\vec{r}$ of all particles at a given time. The two-particle correlation functions:

$$\begin{aligned} D_{x\pm}(R,\tau) &= \langle \frac{1}{2}[x_i(\tau) \pm x_j(\tau)]^2 \delta(R - R_{ij})\rangle_{i\neq j} \\ D_{y\pm}(R,\tau) &= \langle \frac{1}{2}[y_i(\tau) \pm y_j(\tau)]^2 \delta(R - R_{ij})\rangle_{i\neq j} \end{aligned} \quad (1)$$

denote the four eigenmodes corresponding to pairwise motion in 2D. Two of these modes are parallel motions (+) in the longitudinal direction (x) and transverse direction (y). The other two are anti-parallel motions (-) along x and y. By examining the above, it can be seen that such a drift will not affect the anti parallel correlations, but will increase the measured value for the parallel correlation functions D_{x+} and D_{y+}. If $\vec{v}$ was time-independent, then such drift is straightforward to detect and remove from the particle trajectories. However, we often find that $\vec{v}(t)$ has a slow but nontrivial time dependence, and this then makes its influence on D_{x+} and D_{y+} depend on τ. Moreover, due to hydrodynamic interactions, particle motions should have long-range correlations even in the absence of flow, so there is a danger that by removing correlated motion of all the particles, some of the signal from hydrodynamic correlations is lost. As already mentioned, the negative correlations are not affected at all, as they measure the relative displacement of particles, and any center of mass motion cancels out. Likewise, single particle measurements of $\langle\Delta r^2\rangle$ are barely affected by slow drift; to be safe, we calculate our single particle data using $\langle(\Delta\vec{r} - \langle\Delta\vec{r}\rangle)^2\rangle = \langle\Delta r^2\rangle - \langle(\Delta r)^2\rangle$.

To deal with the effects of drift on D_{x+} and D_{y+}, we compute $\langle\Delta\vec{r}(t)\rangle$ at every time t using displacements with lag time 1/30 s, the time between images. We then integrate $\langle\Delta\vec{r}(t)\rangle$ to get a trajectory of the center of mass of all of the particles, $\vec{r}(t)$. Next, we smooth this with a running average over T time steps. We then subtract the smoothed $\vec{r}(t)$ from each individual particle trajectory, to bring the individual particle trajectories into the moving reference frame. In some cases we do not do this smoothing, corresponding to the $T = 1$ limit where the center of mass is forced to be motionless once the trajectories are brought into the moving reference frame.

Figure 1 shows our analysis of how T affects two particle correlations, for several choices of τ. All values of the smoothing parameter T and lag time τ are given in terms of the frame rate of the camera, so $\tau = 1$ corresponds to 1/30 s for example. We desire that our results should be τ independent ideally. Indeed, as should be, the D_{x-}/τ data all collapse for all smoothing parameters T and lag times τ, as mathematically our procedure leaves the anti parallel correlations unchanged. These are the lower curves in Fig. 1 that increase for $R > 2$ μm.

For $T = 1$ in Fig. 1(a), the positive correlations D_{x+} have very slight τ dependency, for lower τ. This is likely due to artificial subtraction of positive correlations as explained above. For $T = 8$ in Fig. 1(b), the positive correlations at higher $\tau = 8, 12, 16$ almost collapse, with slight τ dependence but the positive correlations curve for $\tau = 4$ is much higher. This is due to lack of drift subtraction for $\tau < 8$ when $T = 8$. This is evidence that $\vec{v}(t)$ changes even on a fairly quick time scale of $\tau = 4$ (corresponding to 4/30 s).

Figure 1(c) looks at different smoothing parameters $T = 1, 2, 4, 8$, for the same lag time $\tau = 4$. The curves do not collapse on each other for reasons explained above. Fig. 1(d) looks at the same as data (c), but for $\tau = 12$. The curves for different T collapse nicely on top of each other for $\tau = 12$. While the D_{x+} still have very slight dependence, it is much smaller for $T < \tau$. Hence, at higher lag times τs, we are confident that smoothing does not unduly affect our results, as long as the smoothing parameter T is chosen to be shorter than τ. In order for uniform treatment of all samples, we analyze all movies using $T = 1$. For each movie, we average the data over a wide range of τ's. The smallest τ is always 2 video

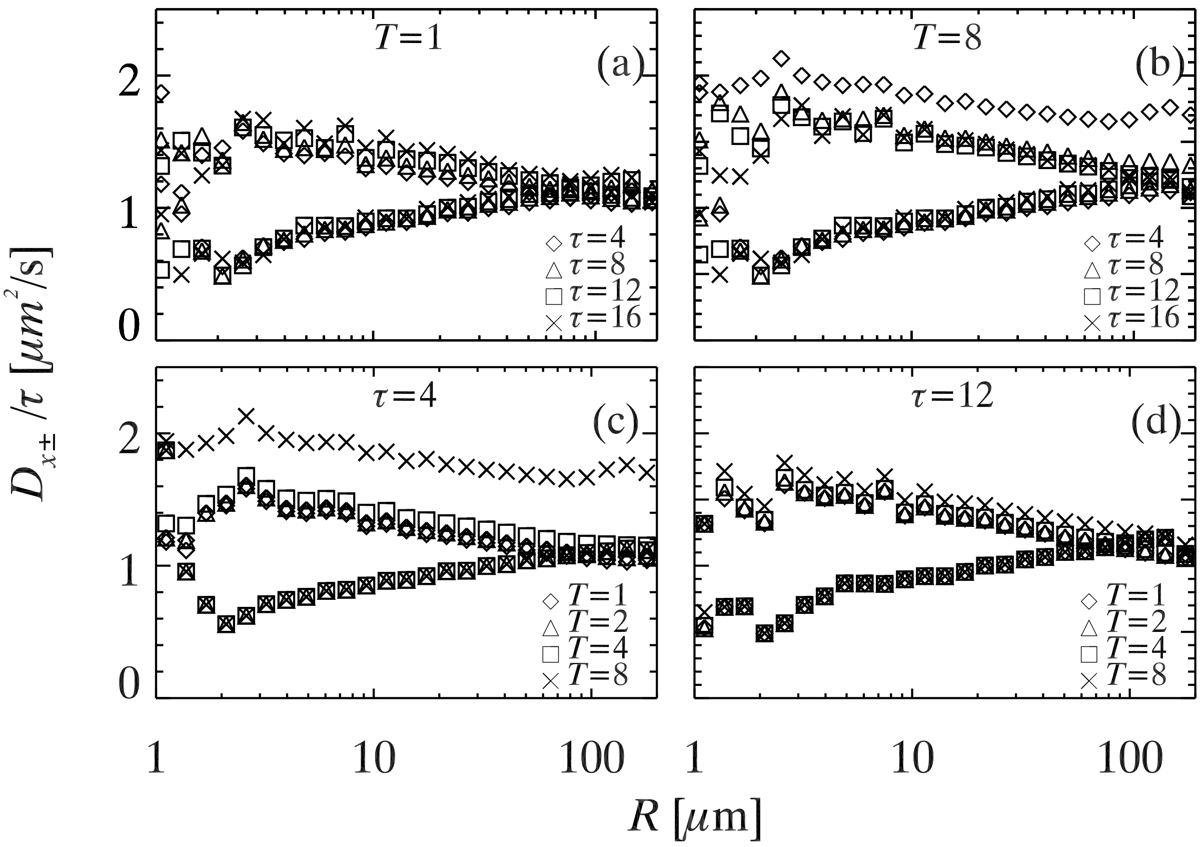

Figure 1. (a) shows D_{x+} and D_{x-} for T=1. (b) shows the same data as (a) for $T = 8$. (c) and (d) show D_{x+} and D_{x-} for a particular $\tau = 4, 12$ respectively. Note that T and τ are in units of video frames, where 1 frame = 1/30s.

frames (66 ms). The largest τ is chosen for each individual movie to be the largest one for which data are available, in other words, the largest duration over which individual particles are tracked; this is at most 1 s. We compute Eqns. 1 for each τ, fit to find the parameters A, B, and L, and then average those parameters over the different τ's. The standard deviations of those data lead to the uncertainties shown in the manuscript. While the evidence of Fig. 1 gives support for our choice $T = 1$, we note that there is still some slight τ dependence, causing larger positive correlations corresponding to larger τ. As only the positive correlations are affected, we would expect A to be different as compared to single particle results. However, our results show that this method works well. As noted in the manuscript, we find $A/D_{1p} = 2.02 \pm 0.12$, which is in good agreement with the expected result $A/D_{1p} = 2$.
